# Retraction: TOX Acts an Oncological Role in Mycosis Fungoides

**DOI:** 10.1371/journal.pone.0283114

**Published:** 2023-05-04

**Authors:** The PLOS ONE Editors

The *PLOS ONE* Editors retract this article [1] because of the following issues.

This article [1] was identified as one of a series of submissions for which we have concerns about peer review, authorship, and similarities across articles. These concerns call into question the validity and provenance of the reported results.

Among other issues, concerns were raised about reuse of image data in Figs 6A and 7D of [1], and about image similarities across articles including:

- between Fig 7D of [1], Fig 3c of [2, 3], Fig 4f of [4], Fig 4D of [5, 6], Fig 3E of [7, 8], and Fig 5E of [9];
- between Fig 6A of [1], Fig 3d of [4], Fig 2E of [9], Fig 2D of [10, 11], and Fig 2b of [12]; and
- between Fig 6B of [1] and Fig 2E of [9].

We regret that the issues were not addressed prior to the article’s publication.

YL, JL, YL, and QS either did not respond directly or could not be reached. XY responded but expressed neither agreement nor disagreement with the editorial decision.

At the time of retraction, the article [1] was republished to address an issue with a data table.
